# Leave-Taking

**Published:** 1883-06

**Authors:** 


### LEAVE-TAKING.

Not all have learned the fine art of leave-taking in an appropriate manner. When you are about to depart, do so at once, gracefully and politely, with no dallying. Don't say "It's about time I was going," and then settle back and talk on aimlessly for another ten minutes. Some people have just such a tiresome habit. They will even rise, and stand about the room in various attitudes, keeping their hosts also standing, and then by an effort succeed in getting as far as the hall, when a new thought strikes them. They brighten up visibly and stand for some minutes longer, saying nothing of importance, but keeping every one in a restless, nervous state. After the door is opened the prolonged leave-taking begins, and everybody in general and in particular is invited to call. Very likely a last thought strikes the departing visitor which his friend must risk a cold to hear to the end. What a relief when the door is finally closed! There is no disguising the fact that they are truly glad he has at last gone. Don't go that way. Make your leave-taking generally short. There is no need of being offensively abrupt, but when you are ready to go—go.

---

Shaved in Seven Kingdoms.—A traveler writes: "I have now been shaved in seven kingdoms and six languages. They all perform the ceremony differently. But they all, from Scotland to Naples, insist on seating you in a plain, straight chair, and bending your head over your back till your spine howls in agony. And they all agree in another custom—they never wash off the soap they have put on. But they bring you a bowl of water, hold it under your chin as you are leaning back, and insist on you washing your own face then and there. If you object to the attitude, they shrug all the upper part of themselves and sling a disdainful smile at you; if you comply, little rivulets run pleasantly down inside of your shirt, and some of the soap they have generously swoggled into your ears gets into your stockings. I have seen no barber wash his victim's face since I landed at Glasgow. Prices vary. In London they charged a shilling (twenty-five cents) for a shave; in Naples they will for fifty centimes (ten cents) shave you, cut your hair, wash your face and hands, curl your eyebrows and wax your mustache till you look like Victor Emmanuel, and can pass for a prince on any of the side streets. Yesterday I was shaved for ten centimes—about two American cents—but I took the balance out in garlic, of which I had a generous bath in the form of respiration. In Verona, the city of the loved and loving Juliet, the barber asked me if I would have my feet washed and my toe-nails cut! That, certainly. is going to extremes."

